# Sentinel Lymph Node Mapping Without Intraoperative Frozen Section in Preoperative Endometrial Intraepithelial Neoplasia/Atypical Endometrial Hyperplasia: Feasibility, Diagnostic Yield, and the Estimated Consequence of Frozen-Section Triage

**DOI:** 10.3390/curroncol33090570

**Published:** 2026-09-21

**Authors:** Mehmet Sait Bakır, Hasan Turan, Osman Doğan, Mürşide Çevikoğlu Kıllı, Numan Bilgiç, Şahin Yüksek

**Affiliations:** Division of Gynecologic Oncology, Department of Obstetrics and Gynecology, Mersin City Training and Research Hospital, 33240 Mersin, Türkiye; hasanturan@gmail.com (H.T.); dogano61deniz@gmail.com (O.D.); mursidecevikoglu@hotmail.com (M.Ç.K.); drnumanbilgic@gmail.com (N.B.); sahindr81@gmail.com (Ş.Y.)

**Keywords:** endometrial intraepithelial neoplasia, endometrial hyperplasia, sentinel lymph node, endometrial neoplasms, frozen sections, lymphatic metastasis, ultrastaging

## Abstract

Background: Endometrial intraepithelial neoplasia/atypical endometrial hyperplasia (EIN/AEH) carries a substantial risk of concurrent endometrial carcinoma at hysterectomy. Sentinel lymph node (SLN) mapping requires cervical injection into an intact uterus, so the decision to map must be made before uterine pathology is known. A strategy that defers that decision to intraoperative frozen section therefore does not choose between mapping and no mapping; it forecloses mapping altogether. We evaluated the technical feasibility and diagnostic yield of SLN mapping performed without frozen section in patients with preoperative EIN/AEH and estimated how much of the nodal disease identified might not have been detected under a frozen-section-guided pathway. Methods: In this retrospective single-center study, 86 patients with a preoperative diagnosis of EIN/AEH underwent hysterectomy, bilateral salpingo-oophorectomy, and SLN mapping with methylene blue between November 2021 and May 2026. No patient underwent intraoperative frozen section, and all SLNs underwent ultrastaging. Patients were grouped by final histopathology, and carcinomas were staged according to FIGO 2023. A counterfactual analysis applied the Mayo intraoperative triage criteria to the final uterine pathology and considered two mechanisms separately: patients who would not have undergone nodal assessment at all, and patients who would have undergone lymphadenectomy but whose disease volume might not have been detected by conventional nodal pathology. Results: Final pathology showed persistent EIN/AEH in 37 patients (43.0%) and endometrial carcinoma in 49 (57.0%). Patients upgraded to carcinoma were older (60.3 ± 9.5 vs. 53.1 ± 10.4 years, *p* = 0.001), more often postmenopausal (87.8% vs. 54.1%, *p* = 0.001), and had greater endometrial thickness (18.6 ± 8.7 vs. 13.5 ± 5.4 mm, *p* = 0.004). At least one SLN was retrieved in 82 of 86 patients (95.3%) and bilaterally in 76 (88.4%); bilateral detection did not differ significantly between groups (94.6% vs. 83.7%, *p* = 0.177). SLNs lay in the obturator region in 62.2% of hemipelves and in the external iliac region in 26.3%, together accounting for 88.5%. Nodal involvement was identified in 11 patients (12.8% of the cohort; 22.4% of carcinomas): isolated tumor cells in two, micrometastasis in six, and macrometastasis in three. Among the patients who underwent additional non-sentinel nodal assessment, no metastatic non-SLN was identified in the presence of negative SLNs; no reference standard was applied to the remaining SLN-negative patients, so a false-negative rate cannot be estimated. No patient with EIN/AEH-only pathology had nodal involvement. Under simulated frozen-section triage, 2 of the 11 node-positive patients (18.2%) would not have undergone nodal assessment at all; a further 6 had low-volume disease that might not have been identified by conventional nodal pathology. Taken together, up to 8 of 11 (72.7%), and up to 6 of the 9 with micro- or macrometastasis (66.7%), might have remained undetected under the specified hypothetical pathway. Conclusions: SLN mapping performed before hysterectomy and without intraoperative frozen section was technically feasible with methylene blue alone and identified occult nodal disease, a substantial part of which might not have been detected under a frozen-section-guided pathway. These findings establish feasibility and diagnostic yield only: the study has no comparison group and supports no conclusion about survival, morbidity, or cost-effectiveness. Because 43.0% of patients had EIN/AEH-only pathology, mapping should form part of individualized preoperative counseling rather than be applied universally.

## Simple Summary

Endometrial intraepithelial neoplasia and atypical endometrial hyperplasia are precancerous conditions in which hidden endometrial cancer is often found after hysterectomy. Sentinel lymph node mapping, a technique that samples the first lymph nodes reached by tumor drainage, can show whether cancer has spread but must be performed before the uterus is removed. In this study of 86 patients, mapping with methylene blue was successful in most cases. Final pathology showed cancer in 49 patients, and lymph node involvement was found in 11. We then estimated what would have happened had the decision to assess lymph nodes been based instead on examination of the uterus during surgery. Two of these patients would not have had their lymph nodes assessed at all, and a further six had deposits so small that they are unlikely to be found without the detailed examination applied to sentinel nodes. These are estimates rather than observations, since no comparison group was studied. These findings suggest that discussing sentinel lymph node mapping before surgery may help avoid missed staging information, while recognizing that many patients will ultimately have no cancer and may not benefit from mapping.

## 1. Introduction

Endometrial intraepithelial neoplasia/atypical endometrial hyperplasia (EIN/AEH) is the recognized precursor lesion of endometrioid endometrial carcinoma. The International Endometrial Collaborative Group proposed separating benign hyperplasia from precancerous endometrial lesions in order to improve classification and management [1]. The 1994 World Health Organization classification divided endometrial hyperplasia into four groups according to architectural complexity and cytologic atypia [2]; the current classification reduces these to two categories, hyperplasia without atypia and EIN/atypical hyperplasia [3].

The clinical importance of EIN/AEH lies chiefly in the frequency with which concurrent endometrial carcinoma is found at hysterectomy, reported in approximately half of patients in several series [4,5]. Optimal surgical management is therefore contested. In patients who have completed childbearing, EIN/AEH is generally treated by total hysterectomy with or without bilateral salpingo-oophorectomy. Because concurrent carcinoma may be present, and may occasionally be of high-risk histologic subtype, many centers use intraoperative frozen section to decide whether nodal assessment is required [6].

That approach has been questioned on practical grounds: frozen section is not universally available, prolongs operative time, and shows meaningful discordance with final pathology in both grade and depth of myometrial invasion [7]. A more fundamental objection is anatomic. Sentinel lymph node (SLN) mapping depends on injecting tracer into the cervix of an intact uterus; once the uterus has been removed, the uterine lymphatic pathways are disrupted, and reliable mapping is no longer possible. Deferring the decision until after hysterectomy and frozen section is therefore not a choice between mapping and no mapping. It forecloses mapping altogether, leaving comprehensive lymphadenectomy, second-stage restaging surgery, or adjuvant treatment decisions made without nodal information as the only remaining options.

Several groups have examined SLN mapping in EIN/AEH over the past decade. Early series described low nodal metastasis rates and argued against routine lymphadenectomy; a meta-analysis of that literature reported pooled rates of 1.6% across all patients with preoperative EIN/AEH and 3.5% among those with concurrent carcinoma [8], and a subsequent systematic review and meta-analysis of surgical nodal assessment in endometrial hyperplasia reached a similar conclusion [9]. As SLN mapping became a standard staging strategy in endometrial carcinoma [10], better-designed studies revisited the question, and the rates they report vary widely. An Italian multicenter study reported nodal metastasis in approximately 7.6% of patients with EIN/AEH and concurrent carcinoma [11], a Canadian series 3.3% of the whole cohort and 6.3% of those with concurrent carcinoma [12], and a Swedish prospective cohort 6.3% and 13.0%, respectively [13]. At the other end of the range, a single-institution series of 221 patients from Memorial Sloan Kettering Cancer Center identified a positive sentinel node in 1 of the 81 mapped patients upstaged to carcinoma (1.2%) [14], a 2026 Italian multicenter cohort of 411 patients reported SLN metastasis in 2.7% of the mapped cohort and 4.7% of those with occult carcinoma [15], and a 2026 Canadian two-center series found nodal disease in 1 of 78 patients (1.2%), consisting of isolated tumor cells alone [16]. Sentinel node biopsy is already used in an analogous premalignant setting ductal carcinoma in situ of the breast, where occult invasive disease may be present [17].

Three questions nevertheless remain open. First, the use of intraoperative frozen section was variable or unreported in the published cohorts, including the most recent multicenter series [15,16]. Where it has been reported, it was applied to a minority of patients and inconsistently: Mueller et al. performed frozen section in 21 of 60 patients managed without sentinel node removal and in 8 of 161 managed with it [14]. The performance of a frozen-section-independent algorithm, in which mapping is completed before hysterectomy in every patient, has therefore not been characterized. Second, and directly related, no study has estimated how much nodal disease would go undetected if nodal assessment were triaged by frozen section, which is precisely the decision a surgeon faces when a patient with preoperative EIN/AEH is brought to the operating room; recent work has begun to quantify the influence of sentinel node findings on adjuvant treatment [15], but not the consequence of forgoing mapping altogether. Third, the comparative series published to date have used indocyanine green [13,14,15] or have not specified the tracer, leaving the performance of blue dye undefined in centers where neither indocyanine green nor reliable intraoperative frozen section is routinely available.

We therefore evaluated a single institutional protocol in which SLN mapping with methylene blue was performed before hysterectomy, independently of frozen section. Our objectives were (i) to determine technical feasibility, expressed as overall and bilateral detection rates; (ii) to describe the frequency, anatomic distribution, and volume of the nodal disease identified; and (iii) to estimate, by applying established intraoperative triage criteria to the final uterine pathology, how much of that nodal disease might not have been identified under a frozen-section-guided pathway. The analysis is confined to the 86 patients in whom this protocol was in fact applied; they are a subset of the patients presenting with EIN/AEH at our institution during the study period, and the protocol was not applied uniformly to all of them (Section 2.1). We did not set out to compare clinical outcomes between strategies, and this design supports no conclusion about survival, surgical morbidity, or cost-effectiveness.

## 2. Materials and Methods

### 2.1. Study Design and Patient Population

**Figure 1:**
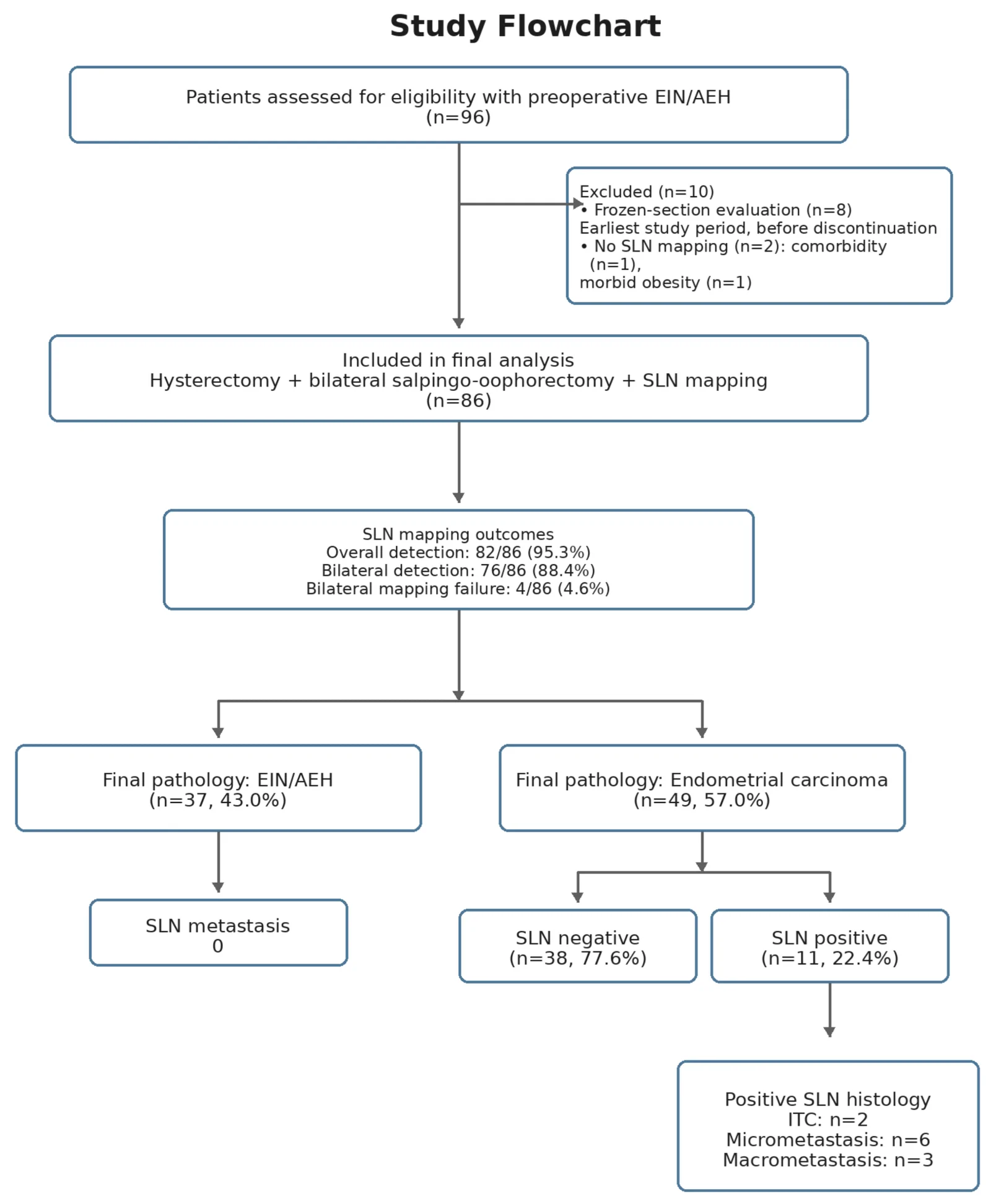
Study flowchart. Of 96 patients with a preoperative diagnosis of EIN/AEH, 8 were excluded because intraoperative frozen-section evaluation was performed (all in the earliest part of the study period, before frozen-section-guided management was discontinued) and 2 because sentinel lymph node mapping was not performed (one for substantial comorbidity, one for morbid obesity), leaving 86 patients for the final analysis. On final histopathology, 37 patients (43.0%) had persistent EIN/AEH and 49 (57.0%) had endometrial carcinoma.

We identified 96 patients with a preoperative diagnosis of EIN/AEH who underwent surgery at Mersin City Training and Research Hospital between November 2021 and May 2026. Eight were excluded because intraoperative frozen section was performed and two because SLN mapping was not attempted, leaving 86 patients for analysis (Figure 1). Patients in whom mapping was attempted but unsuccessful were not excluded and were retained in every denominator. None of the ten excluded patients had endometrial carcinoma on final pathology.

The reasons for exclusion were as follows. Intraoperative frozen section was performed in eight patients, all within the earliest part of the study period, when SLN mapping was being introduced at our institution and frozen-section-guided management remained in use for some patients; the practice was subsequently discontinued. SLN mapping was not attempted in two patients: one with substantial comorbidity in whom operative time was deliberately minimized, and one with morbid obesity in whom the procedure was judged not to be technically feasible. Two consequences of this selection should be noted. Because the eight patients managed with frozen section were drawn from the earliest period, the analyzed cohort is weighted towards the later part of the study period, when the technique was more established, which may favor the reported detection rates. Because none of the 10 excluded patients had carcinoma on final pathology, their exclusion raises the observed rate of concurrent carcinoma from 51.0% across the complete institutional cohort of 96 to 57.0% in the analyzed cohort of 86.

The study period was not an eligibility criterion. Patients were identified by preoperative diagnosis and by the procedures performed; the dates given are the first and last operation dates in the analyzed cohort, taken from the operative records.

The preoperative diagnosis of EIN/AEH was established by endometrial biopsy, hysteroscopy, and/or dilatation and curettage. All patients were assessed preoperatively by gynecologic oncologists and underwent gynecologic examination and transvaginal ultrasonography to evaluate endometrial thickness, suspected myometrial invasion, cervical involvement, and adnexal pathology. Patients with a preoperative diagnosis of endometrial carcinoma, or of hyperplasia without atypia, were excluded. Demographic, clinical, surgical, and pathologic data were collected retrospectively from institutional medical records. The study is reported in accordance with the STROBE statement for observational research.

### 2.2. Surgical Procedure and Sentinel Lymph Node Mapping

All patients underwent hysterectomy, bilateral salpingo-oophorectomy, and SLN mapping. Surgery was performed laparoscopically; patients considered unsuitable for a laparoscopic approach underwent laparotomy. A uterine manipulator was used in 76 patients (88.4%).

Methylene blue dye was injected into the uterine cervix at the three and nine o’clock positions with an insulin syringe, 1 mL superficially and 1 mL deeply at each site. Reinjection was performed when no SLN was identified in one or both hemipelves.

Mapping preceded hysterectomy in every case. After the pelvic sidewall landmarks were exposed, the paravesical and pararectal avascular spaces were developed. Blue-stained lymphatic channels were then followed in the parauterine region, particularly along the uterine artery and the obliterated umbilical artery. In each hemipelvis, the first blue-stained node encountered along the lymphatic pathway was removed and recorded as the sentinel node; any further blue-stained nodes identified along the same channel were also removed and recorded. Any bulky or macroscopically suspicious node was removed irrespective of mapping, and side-specific pelvic lymphadenectomy was performed in any hemipelvis in which no SLN was identified. All procedures were performed by gynecologic oncology surgeons with more than five years’ experience in SLN mapping.

### 2.3. Para-Aortic Assessment

Para-aortic lymphadenectomy was not performed routinely. It was undertaken selectively, as an intraoperative decision, and was performed in one patient in this cohort. That patient had a preoperative diagnosis of EIN. Because of markedly increased endometrial thickness and clinical findings indicating a high probability of malignancy, contrast-enhanced computed tomography of the abdomen and thorax was obtained preoperatively; this reported para-aortic nodes that were indeterminate enlarged but neither bulky nor diagnostic of metastasis with no pelvic nodal abnormality. She was therefore managed under the protocol applied to every patient in this cohort: cervical injection and SLN mapping were performed first, before hysterectomy, and mapping was successful bilaterally. Para-aortic lymphadenectomy was undertaken subsequently, during the same operation, and four para-aortic nodes were involved. The pelvic sentinel node in this patient contained a micrometastasis, that is, disease not macroscopically evident and not shown by the preoperative imaging. Stage IIIC2 was therefore a postoperative pathological assignment and not a preoperative plan. Because para-aortic assessment was selective and imaging-driven rather than systematic, the true rate of para-aortic involvement in this population cannot be determined from these data (Section 4.5).

### 2.4. Data Collection and Verification

Clinical variables comprised age, body mass index, parity, menopausal status, the diagnostic procedure used, and preoperative ultrasonographic endometrial thickness. Surgical variables comprised operative approach, use of a uterine manipulator, adnexal surgery, SLN detection status, side-specific lymphadenectomy, the number of nodes retrieved, and perioperative complications. Pathologic variables comprised histologic type, grade, depth of myometrial invasion, maximum tumor diameter, lymphovascular space invasion (recorded as absent, focal, or substantial), cervical involvement, and nodal status, and were taken from both the preoperative sampling reports and the final hysterectomy pathology reports.

For the present analysis the dataset was rebuilt rather than revised. Every patient was re-identified from the institutional pathology archive by preoperative diagnosis and procedure, and every variable was abstracted afresh from the primary source documents: the preoperative endometrial sampling report, the operative note, the hysterectomy pathology report, the sentinel node pathology report including the ultrastaging levels and immunohistochemistry, the radiology reports, and the multidisciplinary tumor board records. Where a rebuilt value differed from the value previously recorded, the source document was taken as authoritative. Variables that had been incompletely recorded maximum tumor diameter, the focal or substantial character of lymphovascular space invasion, non-SLN status, para-aortic assessment, and perioperative complications were abstracted for the first time. Nodal data were abstracted at the level of the individual node, with the volume of disease and the mode of its detection recorded for each positive node, and the anatomic site recorded for each hemipelvis. Stage was re-derived from the recorded pathological variables rather than transcribed.

### 2.5. Pathologic Evaluation and Sentinel Lymph Node Ultrastaging

All preoperative endometrial samples and hysterectomy specimens were evaluated by gynecologic pathologists with dedicated expertise in gynecologic oncology and in SLN ultrastaging. For the present analysis, the preoperative specimens of every patient with nodal disease were re-reviewed, and the diagnosis of EIN/AEH was confirmed in all cases.

Sentinel lymph nodes were serially sectioned and submitted in their entirety. Every SLN, irrespective of size, underwent the institutional ultrastaging protocol: hematoxylin and eosin (H&E) staining of five sections obtained at 200 µm intervals. Where metastatic disease was evident on H&E, no further staining was required; where the H&E sections were negative, pan-cytokeratin immunohistochemistry was performed to identify micrometastasis or isolated tumor cells. This protocol differs from that of the Memorial Sloan Kettering Cancer Center, in which two levels 50 µm apart are examined [18]; ours examines more levels across a wider span of each block and should therefore be at least as sensitive for low-volume disease. Non-SLNs, those removed at side-specific lymphadenectomy and those removed because they were macroscopically suspicious, were examined with H&E staining alone, without ultrastaging or immunohistochemistry.

For this revision, the mode by which each nodal deposit was first identified, H&E section or pan-cytokeratin immunohistochemistry, was abstracted from the pathology reports and is reported for every patient with nodal involvement in Supplementary Table S1.

Nodal disease was classified according to the American Joint Committee on Cancer definitions applied to axillary nodes in breast cancer: macrometastasis, a deposit larger than 2.0 mm; micrometastasis, a cluster measuring 0.2–2.0 mm; and isolated tumor cells, single cells or clusters measuring 0.2 mm or less and containing fewer than 200 cells [19].

### 2.6. Staging

Carcinomas were staged according to the FIGO 2023 system [20]; FIGO 2009 stages are also reported to permit comparison with earlier series. Three FIGO 2023 rules materially affected our assignments. First, high-grade (grade 3) endometrioid carcinoma is classified, together with serous, clear-cell, mixed, undifferentiated, and carcinosarcoma histologies, as an aggressive histologic type. Second, isolated tumor cells are recorded as pN0(i+) and do not constitute stage IIIC disease, whereas pelvic micrometastasis and macrometastasis correspond to stages IIIC1i and IIIC1ii, respectively. Third, cervical involvement confers stage IIA only where the cervical stroma is invaded; superficial endocervical glandular or mucosal involvement without stromal invasion does not.

FIGO 2009 contains no category for isolated tumor cells, since it predates the routine use of ultrastaging. To avoid attributing to that system a rule it does not contain, we applied the American Joint Committee on Cancer convention consistently to both systems: isolated tumor cells are recorded as pN0(i+) and treated as node-negative under FIGO 2009 as well as under FIGO 2023, and the two patients concerned are staged on uterine factors under FIGO 2009. The number of patients with stage III disease is consequently the same under both systems, and the difference between them in this cohort lies in substage assignment, principally the reclassification of high-grade endometrioid carcinoma as an aggressive histologic type rather than in the number with stage III disease.

Stage was derived for each patient from the recorded pathological variables rather than transcribed from a stage field, and the criterion determining each assignment is given for the 11 patients with nodal involvement in Supplementary Table S1.

### 2.7. Simulated Frozen-Section Triage Analysis

To estimate the diagnostic consequence of a frozen-section-guided strategy, we applied the Mayo intraoperative triage criteria [21] to the final uterine pathology of the 49 patients with carcinoma. Patients with endometrioid histology, grade 1 or 2, myometrial invasion of less than 50%, and a maximum tumor diameter of 20 mm or less were classified as low-risk that is, as patients in whom nodal assessment would have been omitted.

Two mechanisms by which nodal disease might have escaped detection were considered, and they are reported separately throughout because they differ in what they assume.

The first is triage. Patients classified as low-risk would have undergone no nodal assessment at all. This mechanism depends only on the classification rule and on the observed uterine pathology, and the number of node-positive patients it affects is therefore derived directly from the data.

The second is pathologic detection. Patients classified as high-risk would have proceeded to lymphadenectomy, but low-volume nodal disease is identified by serial sectioning and immunohistochemistry of a small number of SLNs and is not routinely sought in the many nodes of a lymphadenectomy specimen. This mechanism depends on an assumption about how a lymphadenectomy specimen would have been examined; the number it affects is therefore an estimate and not an observation.

The two are combined only as an explicitly labeled upper bound. No comparison group was studied, and none of these figures is an observed result.

A further assumption applies to the triage mechanism and should be stated explicitly. The simulation uses final rather than frozen-section pathology and therefore assumes perfect intraoperative assessment. Since frozen section underestimates grade and depth of invasion in a meaningful proportion of cases [7], the analysis is conservative and probably understates how many patients would be triaged away from nodal assessment in practice.

### 2.8. Literature Comparison

To place our findings in context, we performed a structured search of PubMed/MEDLINE and Scopus from database inception to 10 September 2026, using the terms (“sentinel lymph node”) AND (“atypical endometrial hyperplasia” OR “endometrial intraepithelial neoplasia” OR “endometrial hyperplasia with atypia”), without language restriction. We hand-searched the reference lists of the retrieved articles and of the most recent systematic review. Studies were eligible if they reported SLN outcomes in a cohort of at least 50 patients with a preoperative diagnosis of EIN/AEH and reported both the rate of concurrent carcinoma and the rate of nodal involvement with the denominator stated for the patients who actually underwent sentinel node assessment. Reviews, case reports, conference abstracts without full-text publication, and reports of overlapping cohorts from the same institution were excluded; where cohorts overlapped, the most recent or most complete report was retained. Two authors screened records independently and extracted data onto a predefined form, resolving disagreements by discussion. Excluded reports included an administrative-database analysis of nodal assessment in atypical endometrial hyperplasia that reported uptake and perioperative outcomes without pathological detail, as well as a series in which nodal assessment was performed in only part of the cohort and nodal positivity was reported with the whole cohort rather than the assessed patients as denominator, so that its figures are not comparable with those of the other series. Because relevant reports may appear during data collection, manuscript preparation and peer review, the search was not relied upon as originally performed but was re-run immediately before resubmission; the date given above is that of the final search, and the eligible studies it identified have been incorporated the Discussion. This search was undertaken to construct the descriptive comparison presented in Table 6 and was not intended as a systematic review or meta-analysis.

### 2.9. Statistical Analysis

Data were analyzed with SPSS version 23 (IBM Corp., Armonk, NY, USA). Continuous variables are presented as mean ± standard deviation, or as median with range or interquartile range, according to distribution; categorical variables are presented as frequencies and percentages. Patients were grouped by final pathology as persistent EIN/AEH or endometrial carcinoma. Between-group comparisons used the independent-samples *t*-test or the Mann–Whitney U test for continuous variables, and the chi-square test or Fisher’s exact test for categorical variables. A two-sided *p* value below 0.05 was considered significant.

Two denominator conventions were applied. Detection rates used the full analyzed cohort (*N* = 86), including patients in whom mapping was unsuccessful, so that technical success would not be overestimated. SLN histologic status used the 82 patients from whom at least one SLN was retrieved, since nodal status could not be assigned by SLN assessment in the remaining four.

Oncologic outcomes were not analyzed because follow-up was too short to support any inference about recurrence or survival (Section 3.7).

## 3. Results

### 3.1. Cohort Characteristics and Concurrent Carcinoma

Eighty-six patients were analyzed. Final histopathology showed persistent EIN/AEH in 37 (43.0%) and endometrial carcinoma in 49 (57.0%).

The mean age of the cohort was 57.3 ± 10.4 years. Patients with carcinoma were significantly older than those with persistent EIN/AEH (60.3 ± 9.5 vs. 53.1 ± 10.4 years, *p* = 0.001) and more often postmenopausal (43/49, 87.8% vs. 20/37, 54.1%; *p* = 0.001); 63 patients (73.3%) were postmenopausal overall. Mean body mass index was similar in the two groups (33.5 ± 6.7 vs. 32.9 ± 6.4 kg/m^2^, *p* = 0.723), whereas mean preoperative endometrial thickness was significantly greater in the carcinoma group (18.6 ± 8.7 vs. 13.5 ± 5.4 mm, *p* = 0.004). The preoperative diagnosis was obtained by curettage in 72 patients (83.7%) and by hysteroscopy in 14 (16.3%), a distribution that did not differ significantly between groups (*p* = 0.257). Most patients underwent laparoscopic surgery (78/86, 90.7%), and methylene blue was used in every case (Table 1).

### 3.2. Uterine Pathology and Stage

Among the 49 patients with carcinoma, endometrioid carcinoma was the most common histologic type (42 patients, 85.7%); the non-endometrioid histologies were serous carcinoma in 2 (4.1%), clear-cell carcinoma in 2 (4.1%), and mixed carcinoma in 3 (6.1%). Tumor grade was 1 in 23 patients (46.9%), 2 in 14 (28.6%), and 3 in 12 (24.5%). Of the 12 grade 3 tumors, 7 were endometrioid and 5 non-endometrioid; the remaining 2 non-endometrioid tumors were grade 1. Applying the FIGO 2023 definition, 14 patients (28.6%) therefore had an aggressive histologic type: 7 with high-grade endometrioid carcinoma and 7 with non-endometrioid histology.

Myometrial invasion was less than 50% in 27 patients (55.1%) and 50% or greater in 22 (44.9%). Lymphovascular space invasion was present in nine patients (18.4%), focal in four and substantial in five. Cervical involvement was present in four patients (8.2%): stromal invasion in three (6.1%) and superficial endocervical glandular involvement without stromal invasion in one (2.0%). Median maximum tumor diameter was 25 mm (range 5–70), and tumors were larger in patients with nodal involvement than in those without (39.5 ± 19.7 vs. 26.2 ± 14.9 mm, *p* = 0.019).

Under FIGO 2023, the stage distribution was IA2 in 20 patients (40.8%), IB in 9 (18.4%), IIB in 2 (4.1%), IIC in 9 (18.4%), IIIC1i in 5 (10.2%), IIIC1ii in 3 (6.1%), and IIIC2 in 1 (2.0%). No patient was assigned to stage IIA: all three patients with cervical stromal invasion also had nodal involvement and were therefore stage IIIC, which takes precedence, while the single patient with endocervical glandular involvement had no stromal invasion and was therefore staged on depth of myometrial invasion alone, as stage IB. Under FIGO 2009, with isolated tumor cells treated as node-negative in accordance with the convention set out in Section 2.6, the corresponding distribution was IA in 25 patients (51.0%), IB in 15 (30.6%), IIIC1 in 8 (16.3%), and IIIC2 in 1 (2.0%) (Table 2). The number of patients with stage III disease is the same under both systems; the systems differ in this cohort in substage assignment rather than in the identification of stage III disease.

### 3.3. Sentinel Lymph Node Detection and Anatomic Distribution

At least one SLN was retrieved in 82 of 86 patients (95.3%). Detection was bilateral in 76 patients (88.4%) and unilateral in 6 (7.0%), and mapping failed in both hemipelves in 4 (4.7%). Reinjection was performed in three patients (3.5%) in whom no SLN was identified in one or both hemipelves at first injection; of these, bilateral detection was ultimately achieved in one, unilateral detection in one, and mapping failed in one.

Bilateral detection was achieved in 35 of 37 patients (94.6%) with final EIN/AEH and in 41 of 49 (83.7%) with carcinoma, a difference that was not statistically significant (odds ratio 3.41, 95% confidence interval 0.68–17.15; Fisher’s exact test, *p* = 0.177). The median number of sentinel nodes removed was 4.5 (range 0–12) across the analyzed cohort of 86 patients, the minimum of zero corresponding to the 4 patients in whom mapping failed bilaterally.

An SLN was identified in 158 hemipelves, and the anatomic location was recorded for 156 of these; the two unrecorded hemipelves belonged to a single patient. Of the 156 hemipelves with recorded data, SLNs lay in the obturator region in 97 (62.2%), the external iliac region in 41 (26.3%), the common iliac region in 14 (9.0%), and the internal iliac region in 1 (0.6%); in 3 (1.9%) the location was described only as pelvic; the obturator and external iliac regions together accounted for 88.5% of mapped hemipelves (Table 3).

### 3.4. Nodal Metastasis

Nodal involvement was identified in 11 patients: 12.8% of the whole cohort, 13.4% of the 82 patients from whom an SLN was retrieved, and 22.4% of the 49 patients with carcinoma. No patient whose final pathology remained EIN/AEH had nodal involvement.

Nodal disease consisted of isolated tumor cells in two patients, micrometastasis in six, and macrometastasis in three, and was bilateral in two. Excluding isolated tumor cells, as FIGO 2023 requires, the rate of nodal metastasis was 9 of 49 carcinomas (18.4%) and 9 of 86 patients (10.5%); restricted to macrometastasis, it was 3 of 49 (6.1%) and 3 of 86 (3.5%) (Table 4). Patient-level data for all 11 patients with nodal involvement are given in Supplementary Table S1.

Both deposits of isolated tumor cells were identified by pan-cytokeratin immunohistochemistry, performed after the H&E sections of all five ultrastaging levels had been reported as negative. The micrometastases and macrometastases were identified on H&E-stained sections obtained through the ultrastaging protocol. The mode of first detection for each patient is given in Supplementary Table S1.

### 3.5. Non-Sentinel Lymph Nodes

Side-specific or bilateral pelvic lymphadenectomy was performed in 10 patients: the 6 with unilateral detection only and the 4 in whom mapping failed bilaterally. Non-SLN metastasis was identified in two patients, one with four involved para-aortic nodes removed at the selective para-aortic lymphadenectomy described in Section 2.3 and one with a single involved node removed because it was macroscopically suspicious. Both of these patients also had positive SLNs.

Among the patients who underwent additional non-sentinel nodal assessment, with no discordant case, a metastatic non-sentinel node in the presence of negative sentinel nodes was identified. Because non-sentinel nodes were removed only from the 10 patients with unilateral mapping or bilateral mapping failure and from patients with macroscopically suspicious nodes, no reference standard was applied to the remaining patients with negative sentinel nodes. A false-negative rate for sentinel node assessment therefore cannot be estimated from these data.

### 3.6. Simulated Frozen-Section Triage

Applying the four Mayo criteria to the final uterine pathology, 13 of the 49 patients with carcinoma (26.5%) would have been classified as low-risk and would not have undergone nodal assessment.

Triage mechanism. Two of these 13 patients had positive SLNs, one with micrometastasis (tumor diameter 18 mm, grade 1, invasion < 50%) and one with isolated tumor cells (15 mm, grade 2, invasion < 50%); both subsequently received concurrent chemoradiotherapy. Under a frozen-section-guided pathway, these 2 patients, that is 2 of the 11 with nodal involvement (18.2%), would not have undergone nodal assessment at all. This figure follows from the classification rule applied to the observed uterine pathology.

Pathologic detection mechanism. The remaining nine node-positive patients would have been classified as high-risk and would have proceeded to lymphadenectomy. Of these, three had macrometastasis, which conventional single-level examination of a lymphadenectomy specimen would be expected to detect. The other six had low-volume disease, only five micrometastases and one deposit of isolated tumor cells identified through ultrastaging and immunohistochemistry of sentinel nodes, which is not routinely applied to lymphadenectomy specimens. Whether these six would have been identified is not determined by our data; it depends on an assumption about pathological practice.

Upper bound. Combining the two mechanisms, up to 8 of the 11 node-positive patients (72.7%) might have remained undetected under the specified hypothetical pathway. Restricted to patients with micrometastasis or macrometastasis, that is, excluding isolated tumor cells, the corresponding figure is up to six of nine (66.7%) (Table 5). No comparison group was studied, and neither figure is an observed result.

Sensitivity to the triage rule. Omitting maximum tumor diameter and applying only histology, grade, and depth of invasion enlarged the low-risk group from 13 patients to 22 (44.9%), but the same 2 node-positive patients fell within it, and both components of the estimate were unchanged. Neither version of the classification separated node-positive from node-negative patients to a statistically significant degree (*p* = 0.703 and *p* = 0.083, respectively).

### 3.7. Adjuvant Therapy and Perioperative Outcomes

All 11 patients with nodal involvement received concurrent chemoradiotherapy. Among the 38 patients with node-negative carcinoma, 26 received no adjuvant therapy, 7 received vaginal brachytherapy, 4 received external beam radiotherapy with brachytherapy, and 1 received concurrent chemoradiotherapy. These data are descriptive, and treatment decisions cannot be causally attributed to the mapping procedure.

One perioperative complication occurred in the cohort: a urinary tract injury involving the bladder, managed by primary repair (1 of 86, 1.2%). The remaining 85 patients were recorded as having no complication, and there were no lymphoceles, cases of symptomatic lymphedema, vascular or intestinal injuries, reoperations, or thromboembolic events.

Median follow-up was 3.3 months (interquartile range 1.2–11.0; range 0.1–50.3), and 18 patients were followed for 12 months or longer. Because follow-up is immature, recurrence and survival data are not reported.

## 4. Discussion

More than half of the patients in this cohort, all of whom had a preoperative diagnosis of EIN/AEH, proved to have endometrial carcinoma on final histopathology. SLN mapping performed before hysterectomy, with methylene blue and without any reliance on intraoperative frozen section, achieved high overall and bilateral detection rates and identified nodal involvement in 11 patients. The principal new observation is an estimate rather than a measurement: under a frozen-section-guided pathway, a substantial part of the nodal disease found in this cohort might not have been identified.

### 4.1. Concurrent Carcinoma and the Nodal Findings in Context

Our rate of concurrent carcinoma, 57.0%, exceeds the 30–50% reported in most previous series [5,8,11], though it is not far outside that range. Referral bias to a tertiary gynecologic oncology center, a predominantly postmenopausal cohort (73.3%), a high mean body mass index, and the predominance of curettage rather than hysteroscopic resection as the diagnostic method [13] probably all contribute. So does the exclusion of 10 patients, none of whom had carcinoma: across the complete institutional cohort of 96 the rate is 51.0%, in line with the literature [4,5,11,13], and the difference between 51.0% and 57.0% is attributable entirely to that exclusion. These figures support the view that EIN/AEH is not a uniformly low-risk diagnosis but a heterogeneous entity carrying a clinically meaningful risk of occult carcinoma.

Our rate of nodal involvement, 22.4% of patients with concurrent carcinoma, is higher than that of most published series and warrants careful interpretation, because the figure most often quoted from the literature is not directly comparable with it. Isolated tumor cells are recorded as pN0(i+) under FIGO 2023 and do not constitute nodal metastasis; excluding them, our rate falls to 18.4%. Restricting the analysis to macrometastasis, the category most likely to be detected by conventional single-level H&E examination and therefore the most comparable across series using heterogeneous pathology protocols, yields 6.1%. That figure aligns closely with the 7.6% reported by Rosati et al. [11] and lies below the 13.0% reported by Hawez et al. among women with concurrent carcinoma [13]. In the meta-analysis of Vieira-Serna et al., the pooled rate of involved SLNs was 1.6% with all patients with preoperative EIN/AEH as denominator and 3.5% among those with carcinoma [8]. Still lower rates have been reported by Mueller et al. (1 of 81 mapped patients with carcinoma, 1.2%) [14], Catozzo et al. (4.7% of patients with occult carcinoma in a 411-patient multicenter cohort) [15] and Dubé et al. (1.2%, isolated tumor cells alone) [16]. Part of this spread is definitional rather than biological: Mueller et al. counted isolated tumor cells as node-negative when calculating positivity [14], as FIGO 2023 now requires and as the second row of Table 4 does for our cohort, whereas other series aggregate them with metastatic disease. Table 6 sets these series alongside the present cohort.

Part of the gap is attributable to the intensity of pathologic assessment: our protocol applied five levels at 200 µm intervals to every SLN without a size threshold, with pan-cytokeratin immunohistochemistry on all H&E-negative nodes, and both deposits of isolated tumor cells were identified by immunohistochemistry alone, a technique not applied to lymphadenectomy specimens in routine practice. Low-volume disease accounted for 8 of our 11 positive cases (72.7%), comparable to Rosati et al., in whom micrometastases represented 75% of positive SLNs [11]. Pathologic protocol cannot, however, be the whole explanation, since Catozzo et al. also applied ultrastaging to the great majority of sentinel nodes and reported 4.7% among patients with occult carcinoma [15]. The uterine risk profile of the carcinoma group contributes as well deep myometrial invasion in 44.9%, grade 3 disease in 24.5%, non-endometrioid histology in 14.3% although part of this is a function of the classification system, since under FIGO 2023 28.6% of our carcinomas fall into the aggressive category. Selection is a further contributor: this is a single tertiary referral center, the cohort is small, and 10 patients managed differently were excluded, so the confidence interval around our 22.4% is wide and overlaps the published range. Misclassification of an occult carcinoma as EIN/AEH is unlikely to account for the rate, since the preoperative specimens of every patient with nodal disease were re-reviewed and the diagnosis confirmed in all cases.

Two patients had isolated tumor cells only, recorded as pN0(i+) in accordance with FIGO 2023 [20]. The classification rule is settled; the therapeutic question is not. Plante et al. concluded that adjuvant treatment should be tailored to uterine factors rather than to isolated tumor cells alone [23], a recent multi-institutional analysis found no independent prognostic effect in intermediate-risk disease [24], and Awada et al. found no survival difference between radiotherapy and chemoradiotherapy in high-intermediate-risk disease with isolated tumor cells [25]; St Clair et al., by contrast, found low-volume disease to be associated with outcomes intermediate between node-negative and macrometastatic disease [26]; the prospective ENDO-ITC study is designed to resolve the question [27]. Practice consequently varies. At our center, these patients are discussed at a multidisciplinary tumor board and adjuvant treatment is recommended on the basis of the combined uterine and nodal findings; both received concurrent chemoradiotherapy. Because a more conservative reading would count them as node-negative, nodal positivity is presented both including and excluding isolated tumor cells throughout (Table 4).

### 4.2. The Estimated Consequence of Frozen-Section Triage

The central argument for mapping before hysterectomy is structural rather than statistical. Cervical injection is impossible once the uterus has been removed, so choosing to defer nodal assessment to frozen section is choosing to forgo SLN mapping. Sullivan et al. emphasized that SLN biopsy cannot be performed after hysterectomy, and that frozen-section triage may commit some patients to unnecessary comprehensive lymphadenectomy while denying others the opportunity for lower-morbidity staging [28]; Matanes et al. similarly argued that routine SLN sampling before hysterectomy removes the dependence on frozen section [22]. Catozzo et al. have since quantified part of what nodal information contributes, reporting that sentinel node status altered the adjuvant treatment decision in half of the patients who received adjuvant therapy, through de-escalation as well as escalation [15].

Our data allow the size of that consequence to be estimated, though not observed, and the two mechanisms must be kept apart because they rest on different footings. The first is triage: 26.5% of the carcinomas would have been classified as low-risk, and 2 of the 11 node-positive patients fall within that group a component that follows directly from the classification rule applied to the observed pathology. The second is pathologic: among patients who would have proceeded to lymphadenectomy, a further six had low-volume disease only, detected by serial sectioning and immunohistochemistry of a small number of sentinel nodes rather than sought in the many nodes of a lymphadenectomy specimen, a component that rests on an assumption about pathological practice and is therefore an estimate. Combined, they give an upper bound of up to 8 of 11 node-positive patients (72.7%) who might have remained undetected under the specified hypothetical pathway.

The estimate proved robust to how the triage rule was specified: omitting tumor diameter enlarged the low-risk group from 13 patients to 22, yet the same 2 node-positive patients fell within it, both having small, superficially invasive, low-grade tumors that any reasonable intraoperative rule would have judged not to require nodal assessment. Neither version of the classification separated node-positive from node-negative patients significantly, which is itself informative: in this population, uterine features performed poorly as a surrogate for nodal status.

Two assumptions should be kept in view. The simulation uses final rather than frozen-section pathology and so assumes perfect intraoperative reporting of grade, depth and diameter, whereas discordance is well-documented [7]; the number triaged away in practice would therefore be higher than we estimate. And the pathologic component rests on the observation that ultrastaging substantially increases the detection of low-volume disease relative to conventional single-level examination [18,26], which is a statement of likelihood rather than certainty.

### 4.3. Technical Performance, Anatomic Distribution, and the Limits of Sentinel Assessment

Our bilateral detection rate of 88.4% compares favorably with the pooled overall and bilateral rates of 89% and 79% reported by Vieira-Serna et al. [8]. This is notable because methylene blue was used in every case, whereas contemporary algorithms generally rely on indocyanine green, which identifies more SLNs than blue dye in cervical and uterine cancers [29] a finding of direct relevance to centers where indocyanine green is unavailable. Bilateral detection was numerically lower in patients ultimately diagnosed with carcinoma, although the difference did not reach significance and the study was not powered for the comparison; older age and postmenopausal status, both more frequent in that group, are established determinants of reduced mapping success, and tumor-related obstruction of the paracervical channels may impair dye transit. Because mapping is least reliable in precisely the patients most likely to benefit from it, we regard the observation as hypothesis-generating.

The anatomic distribution supports the technical validity of the procedure: SLNs lay in the obturator region in 62.2% of hemipelves and the external iliac region in a further 26.3%, together 88.5%, corresponding closely to the upper paracervical lymphatic pathway draining along the obliterated umbilical artery to the obturator and external iliac basins [30], and consistent with the nodes removed having been true SLNs rather than incidentally stained tissue.

Two limits of what sentinel assessment established in this cohort should be stated. First, radiologically abnormal pelvic nodes are a recognized contraindication to relying on sentinel node mapping as the sole staging procedure, because nodal disease may obstruct lymphatic transit and render mapping unrepresentative. One patient in this cohort had indeterminate enlarged but neither bulky nor diagnostic para-aortic nodes on preoperative imaging, with no pelvic abnormality reported; her pelvic sentinel node contained a micrometastasis, that is, disease that the imaging had not shown. Our protocol in any case removes any macroscopically suspicious node irrespective of mapping, so sentinel assessment was not the sole determinant of nodal status in this patient, and excluding her altered no result of the study Second, non-sentinel nodes were removed only from the 10 patients with unilateral mapping or bilateral mapping failure and from patients with macroscopically suspicious nodes. No reference standard was therefore applied to the remaining patients with negative sentinel nodes, and although no discordant case was identified among those assessed, the sensitivity of sentinel node assessment in this cohort cannot be estimated.

### 4.4. Overtreatment and Patient Selection

The principal counterargument to routine SLN mapping in EIN/AEH is overtreatment: in our cohort, 43.0% of patients had EIN/AEH-only pathology and derived no staging benefit. Sullivan et al. reported that among 141 patients with pre-invasive lesions, 36% had carcinoma on final pathology, only 5% met the Mayo criteria for lymph node dissection, and none had positive nodes or nodal recurrence [28]; Vieira-Serna et al. concluded that the low overall metastasis rate argues against routine evaluation of all patients with preoperative EIN/AEH [8]. This must be weighed against what mapping replaces frozen-section-guided lymphadenectomy, delayed restaging surgery, or adjuvant decisions taken without nodal information and against the observed morbidity of the procedure, which in our cohort amounted to a single bladder injury and which recent multicenter and two-center series have likewise found not to differ materially from hysterectomy alone [15,16].

Selection rather than universal application is increasingly emphasized. Abt et al. found an endometrial stripe of 20 mm or more to be associated with approximately double the risk of concurrent carcinoma [31]; Bell et al. found hysterectomy with SLN dissection cost-effective relative to hysterectomy with frozen section at that same threshold, whereas frozen-section-based management was favored when all EIN patients were considered together [32]; and Hawez et al. reported the highest risk among women diagnosed by endometrial biopsy and those with generalized endometrial thickening [13]. Our data point the same way: the carcinoma group was significantly older, more often postmenopausal, and had greater endometrial thickness, and among patients with carcinoma those with nodal involvement had significantly larger tumors (39.5 ± 19.7 vs. 26.2 ± 14.9 mm, *p* = 0.019). Tumor diameter is not available preoperatively but is measurable on frozen section, and its association with nodal disease here is consistent with the endometrial-thickness findings of Abt et al., suggesting that lesion bulk, however estimated, is the most promising single discriminator.

These observations support an individualized algorithm incorporating age, menopausal status, endometrial thickness, sampling method, imaging findings, and institutional resources, consistent with current guidelines [33]. Because the decision must be made before hysterectomy, patients with EIN/AEH should be told preoperatively that final pathology may reveal invasive carcinoma and, occasionally, nodal metastasis; that mapping may provide staging information during the index operation and reduce the need for restaging surgery; and that no direct therapeutic benefit accrues if final pathology shows only EIN/AEH.

### 4.5. Limitations

This study is retrospective and single-center, and is subject to selection bias and to the limitations of documentation collected outside a prospective protocol.

The design includes no comparison group: patients were not allocated to a frozen-section-guided strategy, and our institution does not use intraoperative frozen section in this setting, so surgical morbidity, operative time, avoidance of restaging surgery, and cost could not be compared between strategies.

The estimate of nodal disease that might not have been identified under a frozen-section-guided pathway is a counterfactual derived from final uterine pathology and not an observed comparison; its two components rest on different assumptions, set out in Section 2.7, and the combined figure is an upper bound.

Changes in adjuvant treatment following SLN findings are reported descriptively and cannot be causally attributed to the mapping procedure.

The protocol was not applied to every patient presenting with EIN/AEH during the study period: eight patients managed with intraoperative frozen section, all from the earliest part of the period, and two in whom mapping was not attempted were excluded. The analyzed cohort is therefore weighted towards the later period, when the technique was more established, which may favor the reported detection rates; because none of the excluded patients had carcinoma, the observed rate of concurrent carcinoma is higher in the analyzed cohort (57.0%) than across the complete institutional cohort (51.0%).

Non-sentinel nodes were removed only from patients with unilateral mapping, bilateral mapping failure, or macroscopically suspicious nodes, so no reference standard was applied to the remaining patients with negative sentinel nodes and the false-negative rate of sentinel node assessment cannot be estimated.

The sample size is modest, particularly for subgroup analyses, and the study was not powered for comparative inference. All procedures were performed by experienced gynecologic oncology surgeons at a tertiary referral center, which limits generalizability to lower-volume settings and may contribute, through referral bias, to the high proportion of patients with concurrent carcinoma. Methylene blue was the sole tracer, and results may differ where indocyanine green is standard.

Para-aortic assessment was selective, guided by imaging rather than performed systematically, so the true rate of para-aortic involvement in this population cannot be determined from these data.

Molecular classification, now integral to endometrial cancer risk stratification, was not routinely performed during the study period: mismatch repair status was available in 12 patients and POLE sequencing in none, so no patient could be assigned a complete molecular subgroup [33,34].

Follow-up is short median 3.3 months, with only 18 patients followed for 12 months or longer so no inference about recurrence or survival can be drawn. Larger prospective studies with adequate follow-up will be required to determine whether SLN mapping in EIN/AEH alters oncologic outcomes or is cost-effective.

## 5. Conclusions

Sentinel lymph node mapping performed before hysterectomy and without intraoperative frozen section is technically feasible in patients with preoperative EIN/AEH, achieving a bilateral detection rate of 88.4% with methylene blue alone, and it identifies occult nodal disease in patients ultimately diagnosed with endometrial carcinoma. Under a frozen-section-guided pathway, 2 of the 11 patients with nodal involvement would not have undergone nodal assessment at all, and a further 6 had disease of a volume that might not have been identified by conventional nodal pathology; taken together, up to three quarters of the nodal disease detected here might have remained undetected. These are estimates derived from the uterine pathology under stated assumptions, not observations. These findings establish feasibility and diagnostic yield; they do not establish clinical benefit, and this design supports no conclusion about survival, morbidity, or cost-effectiveness. Because 43.0% of patients had EIN/AEH-only pathology and derived no staging benefit, SLN mapping should not be mandatory for all patients with EIN/AEH. It should instead form part of individualized preoperative counseling, and the variables that distinguished patients with carcinoma in this cohort, older age, postmenopausal status and greater endometrial thickness, are a reasonable starting point for that discussion.

## Figures and Tables

**Table 1:** Clinical and demographic characteristics of patients.

Variable		Overall (*n* = 86)	EIN/AEH (*n* = 37)	EC (*n* = 49)	*p* Value
Age, years, mean ± SD		57.3 ± 10.4	53.1 ± 10.4	60.3 ± 9.5	0.001
BMI, kg/m^2^, mean ± SD		33.2 ± 6.5	32.9 ± 6.4	33.5 ± 6.7	0.723
Menopausal status	Premenopausal	23 (26.7%)	17 (45.9%)	6 (12.2%)	0.001
	Postmenopausal	63 (73.3%)	20 (54.1%)	43 (87.8%)	
Parity, median (range)		2 (0–8)	—	—	
Gravidity, median (range)		3 (0–11)	—	—	
Diabetes mellitus		25 (29.0%)	—	—	
Hypertension		45 (52.0%)	—	—	
Sampling method	Curettage	72 (83.7%)	29 (78.4%)	43 (87.8%)	0.257
	Hysteroscopy	14 (16.3%)	8 (21.6%)	6 (12.2%)	
Endometrial thickness, mm, mean ± SD		16.3 ± 7.7	13.5 ± 5.4	18.6 ± 8.7	0.004
Surgical approach	Laparoscopy	78 (90.7%)	34 (91.9%)	44 (89.8%)	1.000
	Laparotomy	8 (9.3%)	3 (8.1%)	5 (10.2%)	
Uterine manipulator used		76 (88.4%)	34 (91.9%)	42 (85.7%)	0.504
Tracer	Methylene blue	86 (100%)	37 (100%)	49 (100%)	NA
	Indocyanine green	0	0	0	

BMI, body mass index; EC, endometrial carcinoma; EIN/AEH, endometrial intraepithelial neoplasia/atypical endometrial hyperplasia; NA, not applicable; SD, standard deviation.

**Table 2:** Final histopathological features and stage of patients with endometrial carcinoma (*n* = 49).

Parameter	Category	*n* (%)
Histological type	Endometrioid	42 (85.7%)
	Serous	2 (4.1%)
	Clear-cell	2 (4.1%)
	Mixed	3 (6.1%)
Grade	1	23 (46.9%)
	2	14 (28.6%)
	3	12 (24.5%)
Histological category (FIGO 2023)	Non-aggressive	35 (71.4%)
	Aggressive	14 (28.6%)
Myometrial invasion	<50%	27 (55.1%)
	≥50%	22 (44.9%)
Maximum tumor diameter, mm	Median (range)	25 (5–70)
	≤20 mm	21 (42.9%)
	>20 mm	28 (57.1%)
Lymphovascular space invasion	Absent	40 (81.6%)
	Focal	4 (8.2%)
	Substantial	5 (10.2%)
Cervical involvement	Stromal invasion	3 (6.1%)
	Superficial endocervical glandular involvement only	1 (2.0%)
	None	45 (91.8%)
FIGO 2023 stage	IA2	20 (40.8%)
	IB	9 (18.4%)
	IIB	2 (4.1%)
	IIC	9 (18.4%)
	IIIC1i	5 (10.2%)
	IIIC1ii	3 (6.1%)
	IIIC2	1 (2.0%)
FIGO 2009 stage (for comparison)	IA	25 (51.0%)
	IB	15 (30.6%)
	IIIC1	8 (16.3%)
	IIIC2	1 (2.0%)
Nodal status	Negative	38 (77.6%)
	pN0(i+) isolated tumor cells	2 (4.1%)
	Micrometastasis	6 (12.2%)
	Macrometastasis	3 (6.1%)
Adjuvant therapy	None	26 (53.1%)
	Vaginal brachytherapy	7 (14.3%)
	EBRT + brachytherapy	4 (8.2%)
	Concurrent chemoradiotherapy	12 (24.5%)

EBRT, external beam radiotherapy; FIGO, International Federation of Gynecology and Obstetrics.

**Table 3:** Sentinel lymph node detection and anatomic localization.

Parameter	Category	Overall	EIN/AEH	EC
Detection in at least one hemipelvis		82/86 (95.3%)	36/37 (97.3%)	46/49 (93.9%)
Bilateral detection		76/86 (88.4%)	35/37 (94.6%)	41/49 (83.7%)
Unilateral-only detection		6/86 (7.0%)	1/37 (2.7%)	5/49 (10.2%)
Mapping failure (both hemipelves)		4/86 (4.7%)	1/37 (2.7%)	3/49 (6.1%)
Reinjection performed		3/86 (3.5%)	—	—
Sentinel nodes retrieved, median (range) (*N* = 82)		4.5 (0–12)	—	—
**Anatomic localization**	Obturator	97 (62.2%)	—	—
	External iliac	41 (26.3%)	—	—
	Common iliac	14 (9.0%)	—	—
	Internal iliac	1 (0.6%)	—	—
	Pelvic, not further specified	3 (1.9%)	—	—
Sentinel node histological status	Negative	71/82 (86.6%)	36/36 (100%)	35/46 (76.1%)
	Positive	11/82 (13.4%)	0	11/46 (23.9%)
Volume of nodal disease	Isolated tumor cells	2	0	2
	Micrometastasis	6	0	6
	Macrometastasis	3	0	3
Non-sentinel node metastasis	Any	2	0	2
	With negative sentinel nodes	0	0	0

EC, endometrial carcinoma; EIN/AEH, endometrial intraepithelial neoplasia/atypical endometrial hyperplasia.

**Table 4:** Sentinel lymph node positivity stratified by volume of metastatic disease.

Category	Whole Cohort (*n* = 86)	Patients with Concurrent Carcinoma (*n* = 49)
Any nodal involvement (ITC + micrometastasis + macrometastasis)	11 (12.8%)	11 (22.4%)
Nodal metastasis excluding ITC (micrometastasis + macrometastasis)	9 (10.5%)	9 (18.4%)
Macrometastasis only	3 (3.5%)	3 (6.1%)

ITC, isolated tumor cells.

**Table 5:** Simulated frozen-section triage: the two mechanisms by which nodal disease might have escaped detection (*n* = 49 carcinomas; 11 with nodal involvement).

***Panel A: Mayo intraoperative risk classification, all four criteria (endometrioid, grade 1–2, myometrial invasion < 50%, tumor diameter ≤ 20 mm)***						
**Mayo classification**	**Node-Negative**	**ITC**	**Micrometastasis**	**Macrometastasis**	**Total**	***p* Value**
Low-risk-nodal assessment omitted	11	1	1	0	13 (26.5%)	0.703
High-risk-lymphadenectomy performed	27	1	5	3	36 (73.5%)	
***Panel B: Mechanism 1: triage. Depends only on the classification rule applied to the observed uterine pathology.***						
					***n***	**Of the 11 with Nodal Involvement**
Node-positive patients classified as low- risk, who would not have undergone nodal assessment at all					2	18.20%
*Panel C: Mechanism 2: pathologic detection. Depends on an assumption about how a lymphadenectomy specimen would have been examined; an estimate, not an observation.*						
					***n***	**Of the 11 with Nodal Involvement**
Node-positive patients classified as high- risk whose disease was macrometastatic, which conventional single-level examination would be expected to detect					3	27.30%
Node-positive patients classified as high- risk whose disease was low-volume only (5 micrometastases, 1 deposit of isolated tumor cells), which is not routinely sought in a lymphadenectomy specimen					6	54.50%
*Panel D: Upper bound. The two mechanisms combined; not an observed result.*						
				**Primary Analysis**	**Sensitivity Analysis (Tumor Diameter Omitted)**	***p* Value**
Might have remained undetected, of 11 node-positive patients				up to 8 (72.7%)	up to 8 (72.7%)	0.083
Might have remained undetected, of 9 patients with micro- or macrometastasis				up to 6 (66.7%)	up to 6 (66.7%)	

ITC, isolated tumor cells.

**Table 6:** Comparison of sentinel lymph node outcomes in preoperative EIN/AEH across published studies and the present cohort.

Study	Country	Design	Population	Patients	Nodal Assessment	Overall SLN Detection	Bilateral Detection	Concurrent EC	Positive SLN	Volume of Nodal Disease
Touhami et al., 2018 [12]	Canada	Retrospective	AH and AH suspicious for carcinoma	120	SLN mapping ± LND	NA	NA	64/120 (53.3%)	4/120 (3.3%); 4/64 EC (6.3%)	NA
Mueller et al., 2023 [14]	USA	Retrospective, single institution	Premalignant endometrial pathology (EIN/AEH)	221; ICG injected in 185, SLN removed in 161	SLN biopsy with ICG, with ultrastaging	161/185 (87%)	85%	99/221 (45%)	1/81 mapped EC (1.2%)	Micro 1; ITC 1 (counted node-negative)
Matanes et al., 2023 [22]	Canada	Retrospective	EIN	162; SLN attempted in 157	SLN/LND	143/157 (91.1%)	113/157 (71.9%)	61/162 (37.7%)	2/157 (1.2%); 2/61 EC (3.3%)	Micrometastasis: 2
Hawez et al., 2024 [13]	Sweden	Prospective observational	EIN	98; SLN data in 96	Robotic SLN with ICG	93/96 (96.9%)	83/96 (86.5%)	46/98 (47.0%)	6/96 (6.3%); 6/46 EC (13.0%)	ITC/micro/ macrometastasis in detailed cases
Rosati et al., 2024 [11]	Italy	Multicenter retrospective	AEH	268 in SLN group	SLN biopsy with ICG	255/268 (95.1%)	225/268 (84.0%)	163/268 (60.8%)	12/157 mapped EC (7.6%)	ITC 1; micro 9; macro 2
Catozzo et al., 2026 [15]	Italy	Multicenter retrospective	AEH/EIN	411; SLN in 239	SLN biopsy with ICG (98%), with ultrastaging	94% (patient level)	195/224 (87%)	193/411 (47%)	6/224 (2.7%); 4.7% of mapped EC	ITC 1; micro 2; macro 3
Dubé et al., 2026 [16]	Canada	Retrospective, two centers	EIN	107; nodal assessment in 86	SLN dissection ± LND	78/86 (90.7%)	60/86 (69.8%)	41/86 assessed (48%)	1/78 (1.2%)	ITC 1
Present study	Türkiye	Retrospective	EIN/AEH	86	SLN mapping with methylene blue, no frozen section	82/86 (95.3%)	76/86 (88.4%)	49/86 (57.0%)	11/86 (12.8%); 11/49 EC (22.4%); excluding ITC 9/49 (18.4%); macrometastasis only 3/49 (6.1%)	ITC 2; micro 6 (IIIC1i); macro 3 (IIIC1ii)

AEH, atypical endometrial hyperplasia; AH, atypical hyperplasia; EC, endometrial carcinoma; EIN, endometrial intraepithelial neoplasia; ICG, indocyanine green; ITC, isolated tumor cells; LND, lymph node dissection; NA, not available; SLN, sentinel lymph node.

## Data Availability

The data presented in this study are available from the corresponding author upon reasonable request.

## Supplementary Materials

The following supporting information can be downloaded at https://www.mdpi.com/article/10.3390/curroncol33090570/s1, Table S1: patient-level clinical and pathologic data for the 11 patients with nodal involvement, including the mode of first detection of each nodal deposit and the criterion determining each FIGO 2023 stage assignment.
