# Supplementary material for: Sentinel Lymph Node Mapping Without Intraoperative Frozen Section in Preoperative Endometrial Intraepithelial Neoplasia/Atypical Endometrial Hyperplasia: Feasibility, Diagnostic Yield, and the Estimated Consequence of Frozen-Section Triage
Source: Curr Oncol. 2026 Sep 21;33(9):570. doi: 10.3390/curroncol33090570 (PMC13605372; doi:10.3390/curroncol33090570)
Supplement: Supplementary file 1 [file curroncol-33-00570-s001.zip › curroncol-4542157-supplementary.pdf]

Supplementary Table S1. patient-level clinical and pathologic data for the 11 patients with nodal involvement, including the mode of first detection of each nodal deposit and the criterion determining each FIGO 2023 stage assignment.

Case	Age	Histology	Grade	MI	Size (mm)	LVSI	Cx stroma	R SLN site	R SLN	L SLN site	L SLN	Non-SLN+	Highest volume	Mode of first detection	FIGO 2023	Criterion determining FIGO 2023 stage	FIGO 2009	Adjuvant
1	76	Endometrioid	G3	≥50%	70	Focal	Yes	Not mapped	—	Obturator	Macro	No	Macro	H&E	IIIC1ii	Pelvic macrometastasis	IIIC1	CRT
2	75	Endometrioid	G1	≥50%	54	Absent	Yes	Obturator	Micro	Obturator	Neg	Yes (4 PA)	Micro	H&E	IIIC2	Para-aortic nodal metastasis	IIIC2	CRT
3	72	Endometrioid	G3	≥50%	48	Absent	No	External iliac	Neg	Obturator	Macro	No	Macro	H&E	IIIC1ii	Pelvic macrometastasis	IIIC1	CRT
4	65	Clear cell	G3	≥50%	46	Subst.	Yes	Obturator	Micro	Obturator	Micro	No	Micro	H&E	IIIC1i	Pelvic micrometastasis	IIIC1	CRT
5	62	Endometrioid	G3	≥50%	60	Subst.	No	External iliac	Micro	External iliac	Neg	Yes (1 susp.)	Micro	H&E	IIIC1i	Pelvic micrometastasis	IIIC1	CRT
6	60	Endometrioid	G1	≥50%	19	Absent	No	Obturator	Macro	Obturator	Neg	No	Macro	H&E	IIIC1ii	Pelvic macrometastasis	IIIC1	CRT
7	58	Serous	G1	≥50%	50	Focal	No	Obturator	Micro	Obturator	Micro	No	Micro	H&E	IIIC1i	Pelvic micrometastasis	IIIC1	CRT
8	55	Endometrioid	G3	<50%	15	Absent	No	External iliac	ITC	Obturator	Neg	No	ITC	Cytokeratin IHC	IIC	Aggressive histological type (grade 3 endometrioid) with myometrial invasion; ITC recorded as pN0(i+)	IA	CRT
9	54	Endometrioid	G2	<50%	15	Subst.	No	Obturator	ITC	Obturator	Neg	No	ITC	Cytokeratin IHC	IIB	Substantial LVSI in a non-aggressive histological type; ITC recorded as pN0(i+)	IA	CRT
10	53	Endometrioid	G1	<50%	18	Absent	No	Common iliac	Neg	Common iliac	Micro	No	Micro	H&E	IIIC1i	Pelvic micrometastasis	IIIC1	CRT
11	52	Endometrioid	G2	≥50%	40	Subst.	No	Obturator	Micro	Obturator	Neg	No	Micro	H&E	IIIC1i	Pelvic micrometastasis	IIIC1	CRT

Cx stroma, cervical stromal invasion; CRT, concurrent chemoradiotherapy; H&E, hematoxylin and eosin; IHC, immunohistochemistry; ITC, isolated tumor cells; L, left; Macro, macrometastasis; Micro, micrometastasis; MI, myometrial invasion; LVSI, lymphovascular space invasion; Neg, negative; Non-SLN+, metastatic non-sentinel node; PA, para-aortic; R, right; SLN, sentinel lymph node; Size, maximum tumor diameter.
